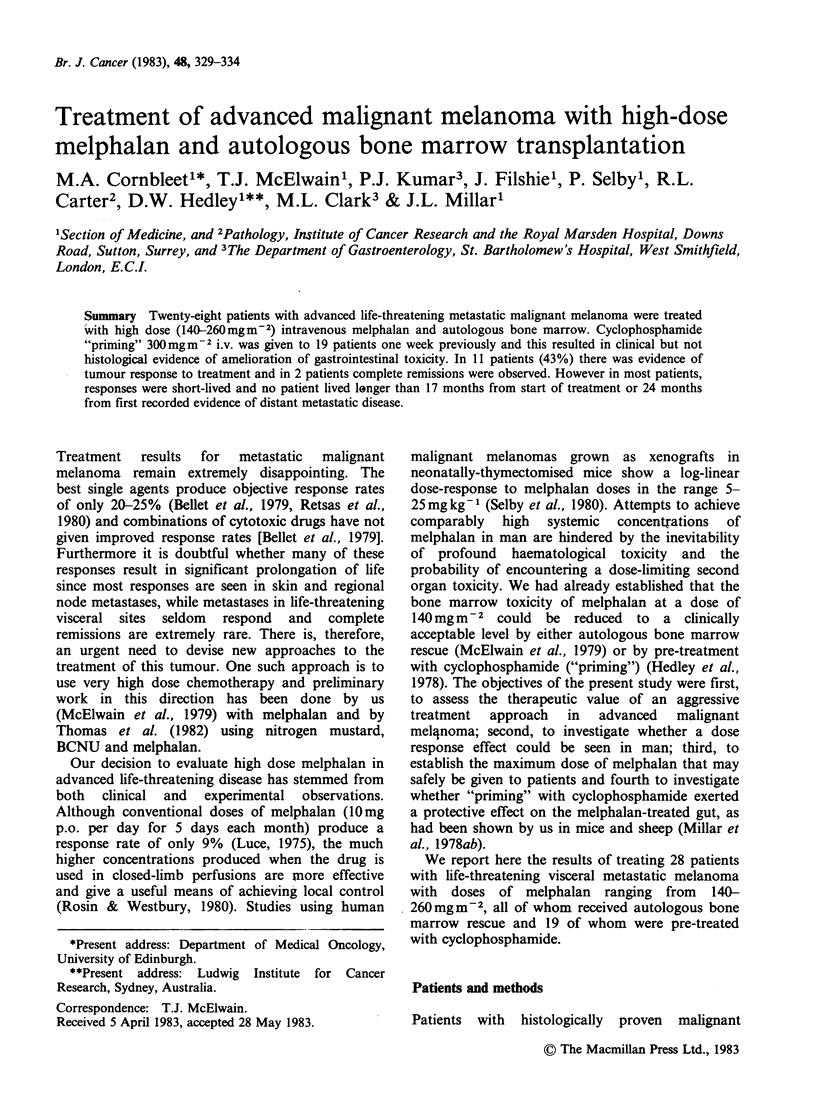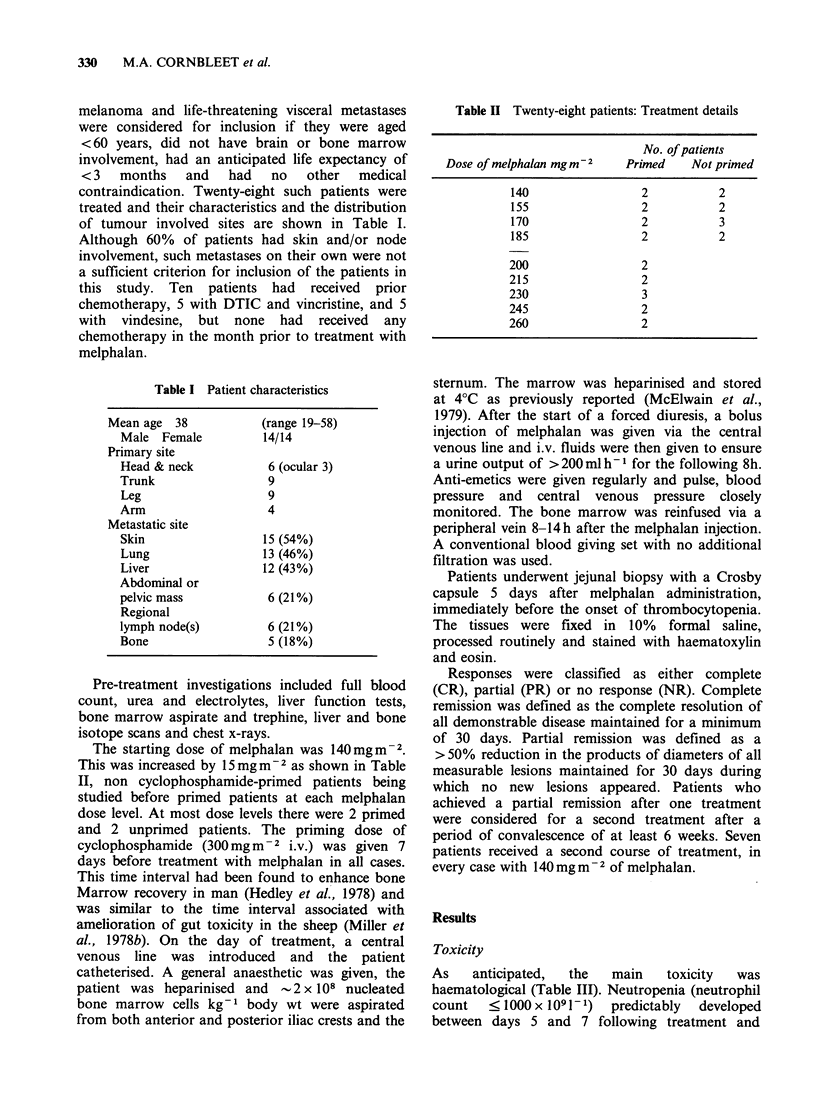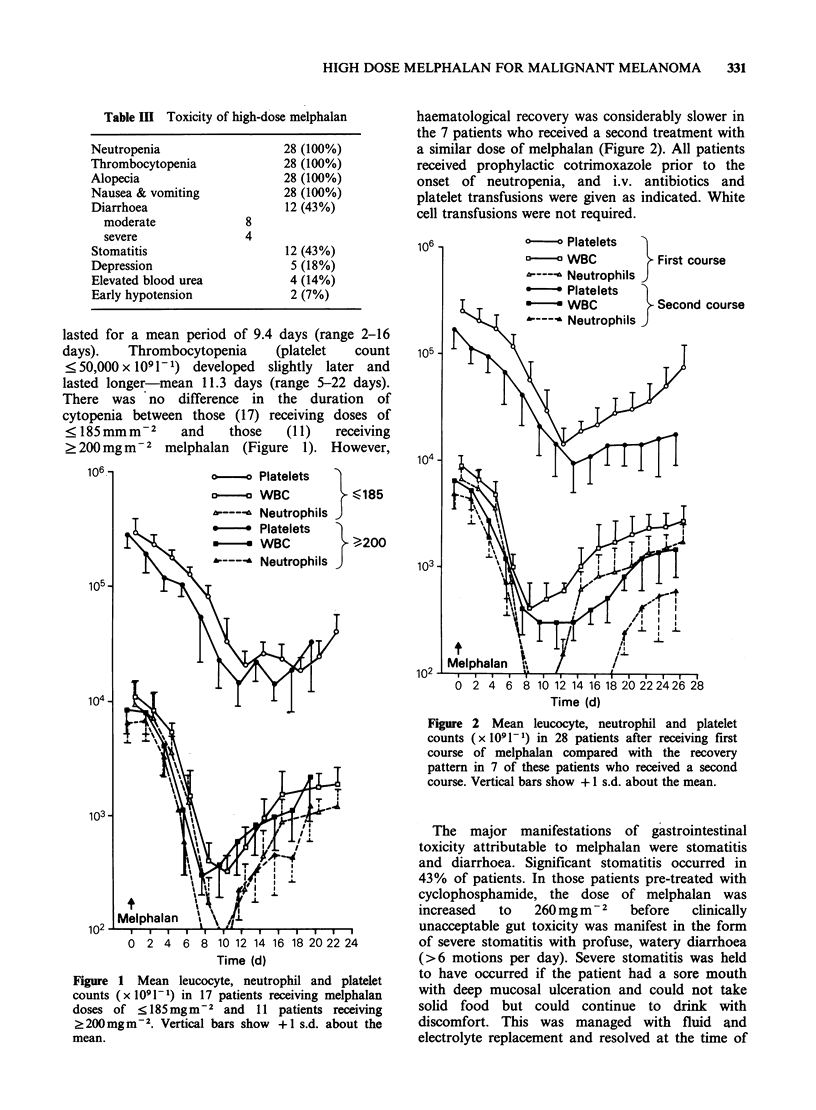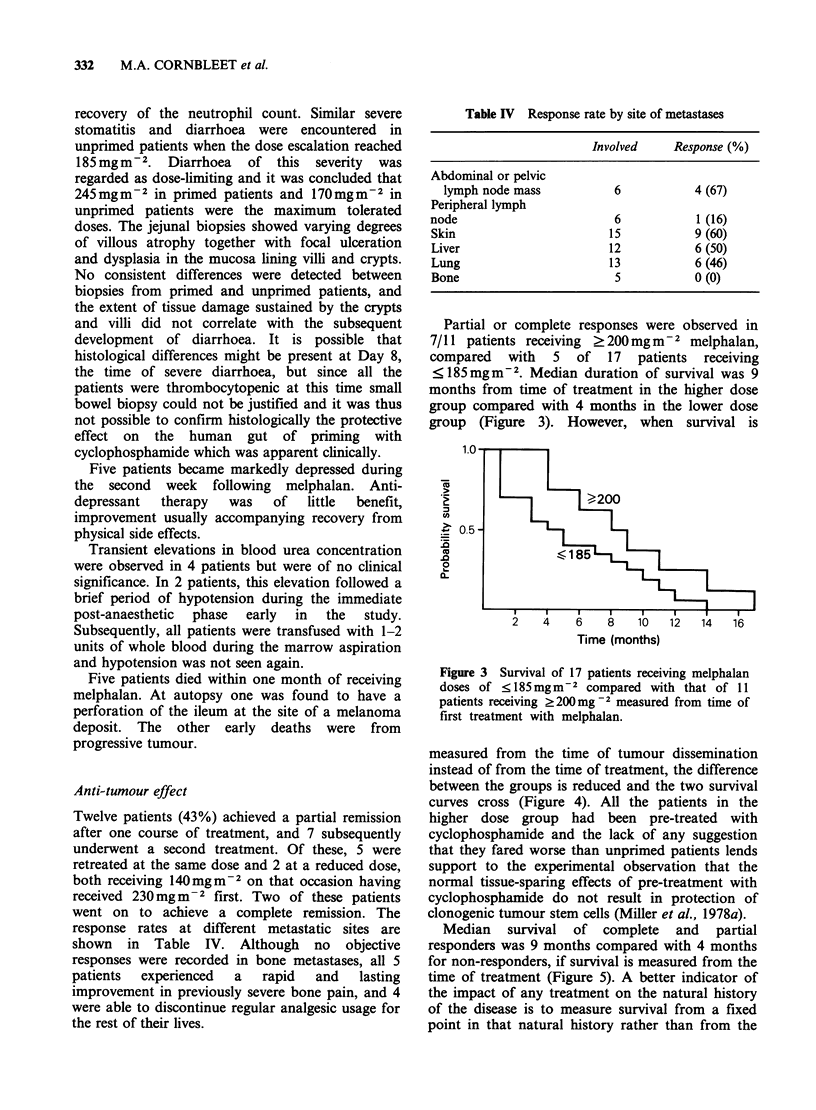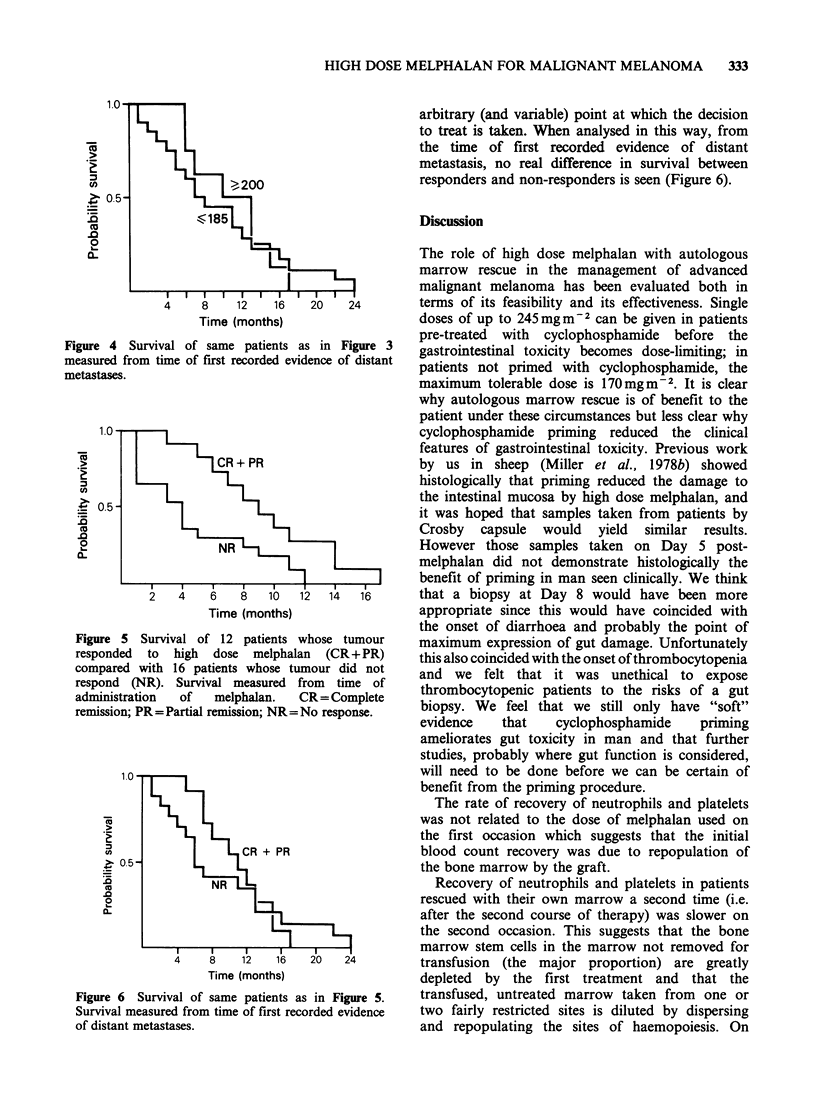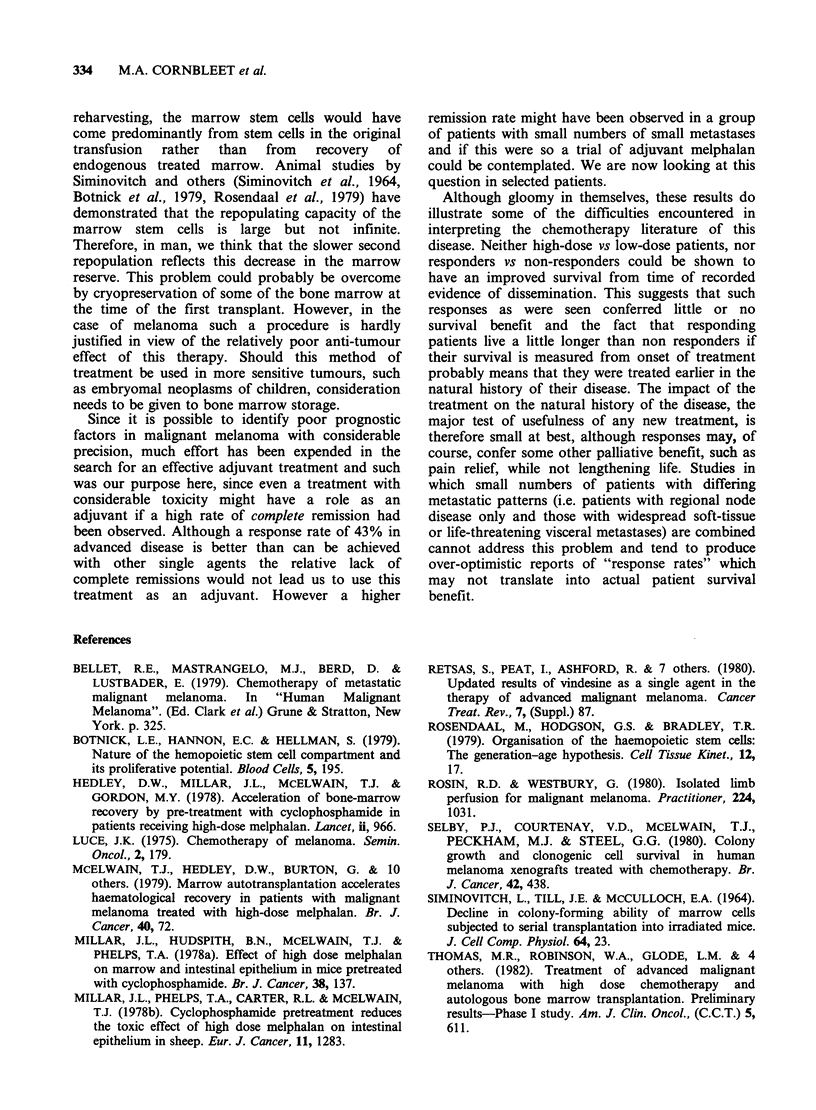# Treatment of advanced malignant melanoma with high-dose melphalan and autologous bone marrow transplantation.

**DOI:** 10.1038/bjc.1983.196

**Published:** 1983-09

**Authors:** M. A. Cornbleet, T. J. McElwain, P. J. Kumar, J. Filshie, P. Selby, R. L. Carter, D. W. Hedley, M. L. Clark, J. L. Millar

## Abstract

Twenty-eight patients with advanced life-threatening metastatic malignant melanoma were treated with high dose (140-260 mgm-2) intravenous melphalan and autologous bone marrow. Cyclophosphamide "priming" 300 mgm-2 i.v. was given to 19 patients one week previously and this resulted in clinical but not histological evidence of amelioration of gastrointestinal toxicity. In 11 patients (43%) there was evidence of tumour response to treatment and in 2 patients complete remissions were observed. However in most patients, responses were short-lived and no patient lived longer than 17 months from start of treatment or 24 months from first recorded evidence of distant metastatic disease.


*Br. J. Cancer* (1983), **48**, 329–334

### Treatment of advanced malignant melanoma with high-dose melphalan and autologous bone marrow transplantation

M.A. Cornbleet[1]*, T.J. McElwain[1], P.J. Kumar[3], J. Filshie[1], P. Selby[1], R.L. Carter[2], D.W. Hedley[1]**, M.L. Clark[3] & J.L. Millar[1]

[1]*Section of Medicine, and* [2]*Pathology, Institute of Cancer Research and the Royal Marsden Hospital, Downs Road, Sutton, Surrey, and* [3]*The Department of Gastroenterology, St. Bartholomew's Hospital, West Smithfield, London, E.C.I.*

**Summary** Twenty-eight patients with advanced life-threatening metastatic malignant melanoma were treated with high dose (140–260 mg m$^{-2}$) intravenous melphalan and autologous bone marrow. Cyclophosphamide "priming" 300 mg m$^{-2}$ i.v. was given to 19 patients one week previously and this resulted in clinical but not histological evidence of amelioration of gastrointestinal toxicity. In 11 patients (43%) there was evidence of tumour response to treatment and in 2 patients complete remissions were observed. However in most patients, responses were short-lived and no patient lived longer than 17 months from start of treatment or 24 months from first recorded evidence of distant metastatic disease.

Treatment results for metastatic malignant melanoma remain extremely disappointing. The best single agents produce objective response rates of only 20–25% (Bellet *et al.*, 1979, Retsas *et al.*, 1980) and combinations of cytotoxic drugs have not given improved response rates [Bellet *et al.*, 1979]. Furthermore it is doubtful whether many of these responses result in significant prolongation of life since most responses are seen in skin and regional node metastases, while metastases in life-threatening visceral sites seldom respond and complete remissions are extremely rare. There is, therefore, an urgent need to devise new approaches to the treatment of this tumour. One such approach is to use very high dose chemotherapy and preliminary work in this direction has been done by us (McElwain *et al.*, 1979) with melphalan and by Thomas *et al.* (1982) using nitrogen mustard, BCNU and melphalan.

Our decision to evaluate high dose melphalan in advanced life-threatening disease has stemmed from both clinical and experimental observations. Although conventional doses of melphalan (10 mg p.o. per day for 5 days each month) produce a response rate of only 9% (Luce, 1975), the much higher concentrations produced when the drug is used in closed-limb perfusions are more effective and give a useful means of achieving local control (Rosin & Westbury, 1980). Studies using human malignant melanomas grown as xenografts in neonatally-thymectomised mice show a log-linear dose-response to melphalan doses in the range 5–25 mg kg$^{-1}$ (Selby *et al.*, 1980). Attempts to achieve comparably high systemic concentrations of melphalan in man are hindered by the inevitability of profound haematological toxicity and the probability of encountering a dose-limiting second organ toxicity. We had already established that the bone marrow toxicity of melphalan at a dose of 140 mg m$^{-2}$ could be reduced to a clinically acceptable level by either autologous bone marrow rescue (McElwain *et al.*, 1979) or by pre-treatment with cyclophosphamide ("priming") (Hedley *et al.*, 1978). The objectives of the present study were first, to assess the therapeutic value of an aggressive treatment approach in advanced malignant melanoma; second, to investigate whether a dose response effect could be seen in man; third, to establish the maximum dose of melphalan that may safely be given to patients and fourth to investigate whether "priming" with cyclophosphamide exerted a protective effect on the melphalan-treated gut, as had been shown by us in mice and sheep (Millar *et al.*, 1978*ab*).

We report here the results of treating 28 patients with life-threatening visceral metastatic melanoma with doses of melphalan ranging from 140–260 mg m$^{-2}$, all of whom received autologous bone marrow rescue and 19 of whom were pre-treated with cyclophosphamide.

### Patients and methods

Patients with histologically proven malignant

*Present address: Department of Medical Oncology, University of Edinburgh.

**Present address: Ludwig Institute for Cancer Research, Sydney, Australia.

Correspondence: T.J. McElwain.
Received 5 April 1983, accepted 28 May 1983.

© The Macmillan Press Ltd., 1983

melanoma and life-threatening visceral metastases were considered for inclusion if they were aged <60 years, did not have brain or bone marrow involvement, had an anticipated life expectancy of <3 months and had no other medical contraindication. Twenty-eight such patients were treated and their characteristics and the distribution of tumour involved sites are shown in Table I. Although 60% of patients had skin and/or node involvement, such metastases on their own were not a sufficient criterion for inclusion of the patients in this study. Ten patients had received prior chemotherapy, 5 with DTIC and vincristine, and 5 with vindesine, but none had received any chemotherapy in the month prior to treatment with melphalan.

**Table I** Patient characteristics

| | |
|---|---|
| Mean age 38 | (range 19–58) |
| Male Female | 14/14 |
| Primary site | |
| Head & neck | 6 (ocular 3) |
| Trunk | 9 |
| Leg | 9 |
| Arm | 4 |
| Metastatic site | |
| Skin | 15 (54%) |
| Lung | 13 (46%) |
| Liver | 12 (43%) |
| Abdominal or pelvic mass | 6 (21%) |
| Regional lymph node(s) | 6 (21%) |
| Bone | 5 (18%) |

Pre-treatment investigations included full blood count, urea and electrolytes, liver function tests, bone marrow aspirate and trephine, liver and bone isotope scans and chest x-rays.

The starting dose of melphalan was $140\,mg\,m^{-2}$. This was increased by $15\,mg\,m^{-2}$ as shown in Table II, non cyclophosphamide-primed patients being studied before primed patients at each melphalan dose level. At most dose levels there were 2 primed and 2 unprimed patients. The priming dose of cyclophosphamide ($300\,mg\,m^{-2}$ i.v.) was given 7 days before treatment with melphalan in all cases. This time interval had been found to enhance bone Marrow recovery in man (Hedley *et al.*, 1978) and was similar to the time interval associated with amelioration of gut toxicity in the sheep (Miller *et al.*, 1978*b*). On the day of treatment, a central venous line was introduced and the patient catheterised. A general anaesthetic was given, the patient was heparinised and $\sim 2 \times 10^8$ nucleated bone marrow cells $kg^{-1}$ body wt were aspirated from both anterior and posterior iliac crests and the

**Table II** Twenty-eight patients: Treatment details

| *Dose of melphalan* $mg\,m^{-2}$ | *No. of patients* *Primed* | *Not primed* |
|---|---|---|
| 140 | 2 | 2 |
| 155 | 2 | 2 |
| 170 | 2 | 3 |
| 185 | 2 | 2 |
| 200 | 2 | |
| 215 | 2 | |
| 230 | 3 | |
| 245 | 2 | |
| 260 | 2 | |

sternum. The marrow was heparinised and stored at 4°C as previously reported (McElwain *et al.*, 1979). After the start of a forced diuresis, a bolus injection of melphalan was given via the central venous line and i.v. fluids were then given to ensure a urine output of $>200\,ml\,h^{-1}$ for the following 8h. Anti-emetics were given regularly and pulse, blood pressure and central venous pressure closely monitored. The bone marrow was reinfused via a peripheral vein 8–14 h after the melphalan injection. A conventional blood giving set with no additional filtration was used.

Patients underwent jejunal biopsy with a Crosby capsule 5 days after melphalan administration, immediately before the onset of thrombocytopenia. The tissues were fixed in 10% formal saline, processed routinely and stained with haematoxylin and eosin.

Responses were classified as either complete (CR), partial (PR) or no response (NR). Complete remission was defined as the complete resolution of all demonstrable disease maintained for a minimum of 30 days. Partial remission was defined as a >50% reduction in the products of diameters of all measurable lesions maintained for 30 days during which no new lesions appeared. Patients who achieved a partial remission after one treatment were considered for a second treatment after a period of convalescence of at least 6 weeks. Seven patients received a second course of treatment, in every case with $140\,mg\,m^{-2}$ of melphalan.

### Results

### *Toxicity*

As anticipated, the main toxicity was haematological (Table III). Neutropenia (neutrophil count $\leq 1000 \times 10^9\,l^{-1}$) predictably developed between days 5 and 7 following treatment and

**Table III** Toxicity of high-dose melphalan

| | | |
|---|---|---|
| Neutropenia | | 28 (100%) |
| Thrombocytopenia | | 28 (100%) |
| Alopecia | | 28 (100%) |
| Nausea & vomiting | | 28 (100%) |
| Diarrhoea | | 12 (43%) |
| moderate | 8 | |
| severe | 4 | |
| Stomatitis | | 12 (43%) |
| Depression | | 5 (18%) |
| Elevated blood urea | | 4 (14%) |
| Early hypotension | | 2 (7%) |

lasted for a mean period of 9.4 days (range 2–16 days). Thrombocytopenia (platelet count $\leq 50{,}000 \times 10^9 l^{-1}$) developed slightly later and lasted longer—mean 11.3 days (range 5–22 days). There was no difference in the duration of cytopenia between those (17) receiving doses of $\leq 185\,mm\,m^{-2}$ and those (11) receiving $\geq 200\,mg\,m^{-2}$ melphalan (Figure 1). However,

**Figure 1** Mean leucocyte, neutrophil and platelet counts ($\times 10^9 l^{-1}$) in 17 patients receiving melphalan doses of $\leq 185\,mg\,m^{-2}$ and 11 patients receiving $\geq 200\,mg\,m^{-2}$. Vertical bars show +1 s.d. about the mean.

haematological recovery was considerably slower in the 7 patients who received a second treatment with a similar dose of melphalan (Figure 2). All patients received prophylactic cotrimoxazole prior to the onset of neutropenia, and i.v. antibiotics and platelet transfusions were given as indicated. White cell transfusions were not required.

**Figure 2** Mean leucocyte, neutrophil and platelet counts ($\times 10^9 l^{-1}$) in 28 patients after receiving first course of melphalan compared with the recovery pattern in 7 of these patients who received a second course. Vertical bars show +1 s.d. about the mean.

The major manifestations of gastrointestinal toxicity attributable to melphalan were stomatitis and diarrhoea. Significant stomatitis occurred in 43% of patients. In those patients pre-treated with cyclophosphamide, the dose of melphalan was increased to $260\,mg\,m^{-2}$ before clinically unacceptable gut toxicity was manifest in the form of severe stomatitis with profuse, watery diarrhoea (>6 motions per day). Severe stomatitis was held to have occurred if the patient had a sore mouth with deep mucosal ulceration and could not take solid food but could continue to drink with discomfort. This was managed with fluid and electrolyte replacement and resolved at the time of recovery of the neutrophil count. Similar severe stomatitis and diarrhoea were encountered in unprimed patients when the dose escalation reached $185 mg m^{-2}$. Diarrhoea of this severity was regarded as dose-limiting and it was concluded that $245 mg m^{-2}$ in primed patients and $170 mg m^{-2}$ in unprimed patients were the maximum tolerated doses. The jejunal biopsies showed varying degrees of villous atrophy together with focal ulceration and dysplasia in the mucosa lining villi and crypts. No consistent differences were detected between biopsies from primed and unprimed patients, and the extent of tissue damage sustained by the crypts and villi did not correlate with the subsequent development of diarrhoea. It is possible that histological differences might be present at Day 8, the time of severe diarrhoea, but since all the patients were thrombocytopenic at this time small bowel biopsy could not be justified and it was thus not possible to confirm histologically the protective effect on the human gut of priming with cyclophosphamide which was apparent clinically.

Five patients became markedly depressed during the second week following melphalan. Anti-depressant therapy was of little benefit, improvement usually accompanying recovery from physical side effects.

Transient elevations in blood urea concentration were observed in 4 patients but were of no clinical significance. In 2 patients, this elevation followed a brief period of hypotension during the immediate post-anaesthetic phase early in the study. Subsequently, all patients were transfused with 1–2 units of whole blood during the marrow aspiration and hypotension was not seen again.

Five patients died within one month of receiving melphalan. At autopsy one was found to have a perforation of the ileum at the site of a melanoma deposit. The other early deaths were from progressive tumour.

### *Anti-tumour effect*

Twelve patients (43%) achieved a partial remission after one course of treatment, and 7 subsequently underwent a second treatment. Of these, 5 were retreated at the same dose and 2 at a reduced dose, both receiving $140 mg m^{-2}$ on that occasion having received $230 mg m^{-2}$ first. Two of these patients went on to achieve a complete remission. The response rates at different metastatic sites are shown in Table IV. Although no objective responses were recorded in bone metastases, all 5 patients experienced a rapid and lasting improvement in previously severe bone pain, and 4 were able to discontinue regular analgesic usage for the rest of their lives.

**Table IV** Response rate by site of metastases

| | *Involved* | *Response* (%) |
|---|---|---|
| Abdominal or pelvic lymph node mass | 6 | 4 (67) |
| Peripheral lymph node | 6 | 1 (16) |
| Skin | 15 | 9 (60) |
| Liver | 12 | 6 (50) |
| Lung | 13 | 6 (46) |
| Bone | 5 | 0 (0) |

Partial or complete responses were observed in 7/11 patients receiving $\geq 200 mg m^{-2}$ melphalan, compared with 5 of 17 patients receiving $\leq 185 mg m^{-2}$. Median duration of survival was 9 months from time of treatment in the higher dose group compared with 4 months in the lower dose group (Figure 3). However, when survival is

**Figure 3** Survival of 17 patients receiving melphalan doses of $\leq 185 mg m^{-2}$ compared with that of 11 patients receiving $\geq 200 mg^{-2}$ measured from time of first treatment with melphalan.

measured from the time of tumour dissemination instead of from the time of treatment, the difference between the groups is reduced and the two survival curves cross (Figure 4). All the patients in the higher dose group had been pre-treated with cyclophosphamide and the lack of any suggestion that they fared worse than unprimed patients lends support to the experimental observation that the normal tissue-sparing effects of pre-treatment with cyclophosphamide do not result in protection of clonogenic tumour stem cells (Miller *et al.*, 1978*a*).

Median survival of complete and partial responders was 9 months compared with 4 months for non-responders, if survival is measured from the time of treatment (Figure 5). A better indicator of the impact of any treatment on the natural history of the disease is to measure survival from a fixed point in that natural history rather than from the

**Figure 4** Survival of same patients as in **Figure 3** measured from time of first recorded evidence of distant metastases.

**Figure 5** Survival of 12 patients whose tumour responded to high dose melphalan (CR+PR) compared with 16 patients whose tumour did not respond (NR). Survival measured from time of administration of melphalan. CR=Complete remission; PR=Partial remission; NR=No response.

**Figure 6** Survival of same patients as in **Figure 5**. Survival measured from time of first recorded evidence of distant metastases.

arbitrary (and variable) point at which the decision to treat is taken. When analysed in this way, from the time of first recorded evidence of distant metastasis, no real difference in survival between responders and non-responders is seen (Figure 6).

### Discussion

The role of high dose melphalan with autologous marrow rescue in the management of advanced malignant melanoma has been evaluated both in terms of its feasibility and its effectiveness. Single doses of up to 245 mg m$^{-2}$ can be given in patients pre-treated with cyclophosphamide before the gastrointestinal toxicity becomes dose-limiting; in patients not primed with cyclophosphamide, the maximum tolerable dose is 170 mg m$^{-2}$. It is clear why autologous marrow rescue is of benefit to the patient under these circumstances but less clear why cyclophosphamide priming reduced the clinical features of gastrointestinal toxicity. Previous work by us in sheep (Miller *et al.*, 1978*b*) showed histologically that priming reduced the damage to the intestinal mucosa by high dose melphalan, and it was hoped that samples taken from patients by Crosby capsule would yield similar results. However those samples taken on Day 5 post-melphalan did not demonstrate histologically the benefit of priming in man seen clinically. We think that a biopsy at Day 8 would have been more appropriate since this would have coincided with the onset of diarrhoea and probably the point of maximum expression of gut damage. Unfortunately this also coincided with the onset of thrombocytopenia and we felt that it was unethical to expose thrombocytopenic patients to the risks of a gut biopsy. We feel that we still only have "soft" evidence that cyclophosphamide priming ameliorates gut toxicity in man and that further studies, probably where gut function is considered, will need to be done before we can be certain of benefit from the priming procedure.

The rate of recovery of neutrophils and platelets was not related to the dose of melphalan used on the first occasion which suggests that the initial blood count recovery was due to repopulation of the bone marrow by the graft.

Recovery of neutrophils and platelets in patients rescued with their own marrow a second time (i.e. after the second course of therapy) was slower on the second occasion. This suggests that the bone marrow stem cells in the marrow not removed for transfusion (the major proportion) are greatly depleted by the first treatment and that the transfused, untreated marrow taken from one or two fairly restricted sites is diluted by dispersing and repopulating the sites of haemopoiesis. On reharvesting, the marrow stem cells would have come predominantly from stem cells in the original transfusion rather than from recovery of endogenous treated marrow. Animal studies by Siminovitch and others (Siminovitch *et al.*, 1964, Botnick *et al.*, 1979, Rosendaal *et al.*, 1979) have demonstrated that the repopulating capacity of the marrow stem cells is large but not infinite. Therefore, in man, we think that the slower second repopulation reflects this decrease in the marrow reserve. This problem could probably be overcome by cryopreservation of some of the bone marrow at the time of the first transplant. However, in the case of melanoma such a procedure is hardly justified in view of the relatively poor anti-tumour effect of this therapy. Should this method of treatment be used in more sensitive tumours, such as embryomal neoplasms of children, consideration needs to be given to bone marrow storage.

Since it is possible to identify poor prognostic factors in malignant melanoma with considerable precision, much effort has been expended in the search for an effective adjuvant treatment and such was our purpose here, since even a treatment with considerable toxicity might have a role as an adjuvant if a high rate of *complete* remission had been observed. Although a response rate of 43% in advanced disease is better than can be achieved with other single agents the relative lack of complete remissions would not lead us to use this treatment as an adjuvant. However a higher remission rate might have been observed in a group of patients with small numbers of small metastases and if this were so a trial of adjuvant melphalan could be contemplated. We are now looking at this question in selected patients.

Although gloomy in themselves, these results do illustrate some of the difficulties encountered in interpreting the chemotherapy literature of this disease. Neither high-dose *vs* low-dose patients, nor responders *vs* non-responders could be shown to have an improved survival from time of recorded evidence of dissemination. This suggests that such responses as were seen conferred little or no survival benefit and the fact that responding patients live a little longer than non responders if their survival is measured from onset of treatment probably means that they were treated earlier in the natural history of their disease. The impact of the treatment on the natural history of the disease, the major test of usefulness of any new treatment, is therefore small at best, although responses may, of course, confer some other palliative benefit, such as pain relief, while not lengthening life. Studies in which small numbers of patients with differing metastatic patterns (i.e. patients with regional node disease only and those with widespread soft-tissue or life-threatening visceral metastases) are combined cannot address this problem and tend to produce over-optimistic reports of "response rates" which may not translate into actual patient survival benefit.

### References

BELLET, R.E., MASTRANGELO, M.J., BERD, D. & LUSTBADER, E. (1979). Chemotherapy of metastatic malignant melanoma. In "Human Malignant Melanoma". (Ed. Clark *et al.*) Grune & Stratton, New York. p. 325.

BOTNICK, L.E., HANNON, E.C. & HELLMAN, S. (1979). Nature of the hemopoietic stem cell compartment and its proliferative potential. *Blood Cells*, **5**, 195.

HEDLEY, D.W., MILLAR, J.L., MCELWAIN, T.J. & GORDON, M.Y. (1978). Acceleration of bone-marrow recovery by pre-treatment with cyclophosphamide in patients receiving high-dose melphalan. *Lancet*, **ii**, 966.

LUCE, J.K. (1975). Chemotherapy of melanoma. *Semin. Oncol.*, **2**, 179.

MCELWAIN, T.J., HEDLEY, D.W., BURTON, G. & 10 others. (1979). Marrow autotransplantation accelerates haematological recovery in patients with malignant melanoma treated with high-dose melphalan. *Br. J. Cancer*, **40**, 72.

MILLAR, J.L., HUDSPITH, B.N., MCELWAIN, T.J. & PHELPS, T.A. (1978a). Effect of high dose melphalan on marrow and intestinal epithelium in mice pretreated with cyclophosphamide. *Br. J. Cancer*, **38**, 137.

MILLAR, J.L., PHELPS, T.A., CARTER, R.L. & MCELWAIN, T.J. (1978b). Cyclophosphamide pretreatment reduces the toxic effect of high dose melphalan on intestinal epithelium in sheep. *Eur. J. Cancer*, **11**, 1283.

RETSAS, S., PEAT, I., ASHFORD, R. & 7 others. (1980). Updated results of vindesine as a single agent in the therapy of advanced malignant melanoma. *Cancer Treat. Rev.*, **7**, (Suppl.) 87.

ROSENDAAL, M., HODGSON, G.S. & BRADLEY, T.R. (1979). Organisation of the haemopoietic stem cells: The generation–age hypothesis. *Cell Tissue Kinet.*, **12**, 17.

ROSIN, R.D. & WESTBURY, G. (1980). Isolated limb perfusion for malignant melanoma. *Practitioner*, **224**, 1031.

SELBY, P.J., COURTENAY, V.D., MCELWAIN, T.J., PECKHAM, M.J. & STEEL, G.G. (1980). Colony growth and clonogenic cell survival in human melanoma xenografts treated with chemotherapy. *Br. J. Cancer*, **42**, 438.

SIMINOVITCH, L., TILL, J.E. & MCCULLOCH, E.A. (1964). Decline in colony-forming ability of marrow cells subjected to serial transplantation into irradiated mice. *J. Cell Comp. Physiol.* **64**, 23.

THOMAS, M.R., ROBINSON, W.A., GLODE, L.M. & 4 others. (1982). Treatment of advanced malignant melanoma with high dose chemotherapy and autologous bone marrow transplantation. Preliminary results—Phase I study. *Am. J. Clin. Oncol.*, (C.C.T.) **5**, 611.